# Supply–demand governance of hierarchical healthcare systems: mobile big data unveils non-random patient flow patterns and the bypass premium in cities

**DOI:** 10.3389/fpubh.2026.1797443

**Published:** 2026-03-11

**Authors:** Qing Guo, Hengna Ren, Xinmiao Shao

**Affiliations:** 1 Business School, University of Shanghai for Science and Technology, Shanghai, China; 2 School of Marxism, Shanghai University of Engineering Science, Shanghai, China

**Keywords:** bypass premium, hierarchical healthcare system, mobile signaling data, null model, patient flow network, spatial equity

## Abstract

**Background:**

The uneven distribution of healthcare resources and jobs-housing spatial separation are reshaping the spatiotemporal patterns of urban patient flows. This structural mismatch exacerbates inequalities in service utilization and imposes hidden geographic and social costs. However, conventional static statistics and theoretical models often fail to capture authentic micro-level behavioral patterns, rendering them unable to precisely quantify or deconstruct the inequalities and burdens concealed within patient flows.

**Methods:**

Taking Shanghai as a representative megacity case study, we utilized anonymized mobile signaling data (March 2019) to construct a weighted, directed “demand–supply” patient flows network. We introduced a null model as a random benchmark and employed the channel decomposition method to deconstruct pathway structures. We developed a “bypass premium” index to quantify the specific burden of quality-driven hospital seeking.

**Results:**

(1) Resource siphoning: Patient flows are highly concentrated toward top-tier hospitals, yet their spatial footprint is widely dispersed across the city, a pattern that deviates significantly from the random benchmark. (2) Boundary filtering: Administrative boundaries act as a “value filter.” Inter-district flows do not diffuse uniformly but are funneled into backbone pathways leading exclusively to tertiary hospitals. (3) Functional neutrality: Secondary hospitals fail to perform their intended hub-and-diversion function within the hierarchical healthcare system, resulting in a state of functional neutrality. (4) Cost deconstruction: The average bypass premium for reaching a tertiary hospital is 10.24 km. Crucially, 73.54% (7.53 km) of this constitutes passive structural friction required to overcome boundary barriers, while only 26.46% (2.71 km) represents the active selective premium paid for quality-driven access.

**Conclusion:**

This study confirms the non-random polarization of patient flows and the screening mechanism of administrative boundaries in Shanghai. Our findings reveal that the costs of inter-district hospital-seeking stem primarily from passive structural friction rather than active selective premiums, occurring alongside a critical functional deficit in secondary hospitals. Consequently, policy interventions must prioritize strategies of “reducing friction” and “strengthening the middle.” Specifically, optimizing transportation networks, insurance integration, and medical consortiums is essential to dismantle barriers and revitalize the hub capacity of the intermediate tier.

## 1 Introduction

Urban healthcare resources are typically clustered in city centers, whereas peripheral areas face relative deficits in resource density and service capacity (1, 2). As population sprawls outward, jobs-housing spatial separation and expanding transportation networks have extended the spatial reach of patient flows (3, 4). Concurrently, driven by the interplay of behavioral preferences and inertia, patients actively bypass administrative boundaries and hierarchical constraints (5), evolving into structured, long-distance hospital-seeking patterns. This spatial mismatch between static resource supply and dynamic patient flows not only triggers systemic inefficiency but also escalates the geographic and social costs of healthcare-seeking (6), thereby compromising the equity and efficiency of the hierarchical healthcare system. Consequently, it is imperative to shift the research lens to micro-level dynamic flows. By characterizing these patterns and deconstructing their spatial structures, this study aims to provide a scientific basis for institutional optimization and rational resource allocation.

Although existing studies provide a solid foundation for understanding the supply–demand matching of urban hierarchical healthcare services, significant limitations remain in diagnosing system efficiency and equity from the perspective of micro-level patient flows. First, traditional research is predominantly limited to unilateral assessments based on static resource supply. Some studies utilize statistical indicators such as the Theil index and Gini coefficient to evaluate quantitative disparities in resource distribution (7, 8), while others employ models like the 2SFCA to quantify spatial accessibility based on static metrics such as population and bed count (9, 10). Crucially, these studies overlook the spatiotemporal characteristics of supply–demand interactions; they neither capture the authentic paths of patient flows nor characterize the dynamic load of hierarchical healthcare resources in reality. Second, due to the scarcity of micro-trajectory data, many studies rely on theoretical models, such as gravity models and radiation models (11, 12), to infer macroscopic population flows. Such models depend heavily on assumptions and parameter settings, making it difficult to precisely capture real-world flow targets and micro-behavioral patterns. Regarding network construction strategies, existing research often employs administrative divisions or grid units to build single-mode networks (13, 14), which blurs the functional distinction between supply and demand units and obscures “point-to-point” supply–demand directionality. Furthermore, while current scholarship reveals the macroscopic morphology of patient flows (15, 16), it rarely deconstructs these macroscopic phenomena into behavioral patterns and trade-off mechanisms at the micro-pathway level. Consequently, existing conclusions struggle to support the refined allocation of hierarchical healthcare resources and institutional optimization.

Against this backdrop, this study endeavors to explore the application paradigm of data-driven technologies in the governance of urban hierarchical healthcare systems. Using Shanghai as a representative empirical case to validate this methodological approach, we utilize mobile signaling data to reveal the hospital-seeking behavioral patterns and trade-off mechanisms of urban residents. Specifically, this study designates public general hospitals in Shanghai as the supply side and jobs-housing locations as the demand side. Using anonymized mobile signaling data from Shanghai in March 2019, we identify hospital-seeking trajectories to construct a city-scale weighted directed patient flow network. Centered on this network, our analysis proceeds in four stages: First, we characterize the global properties of the network to establish a macroscopic understanding of its structural architecture. Second, utilizing a null model as a stochastic benchmark, we test the structural dependence and non-random clustering characteristics of patient flows. Subsequently, through pathway decomposition and the bypass premium metric, we decode the dual constraints of administrative boundary barriers and distance on healthcare-seeking behavior. Finally, based on the empirical findings, we identify governance targets and institutional optimization pathways for the spatial refinement of Shanghai’s hierarchical healthcare resource allocation, offering a data-driven reference for the governance of tiered healthcare systems in other cities.

## 2 Data sources and network construction

### 2.1 Study area

**Figure 1:**
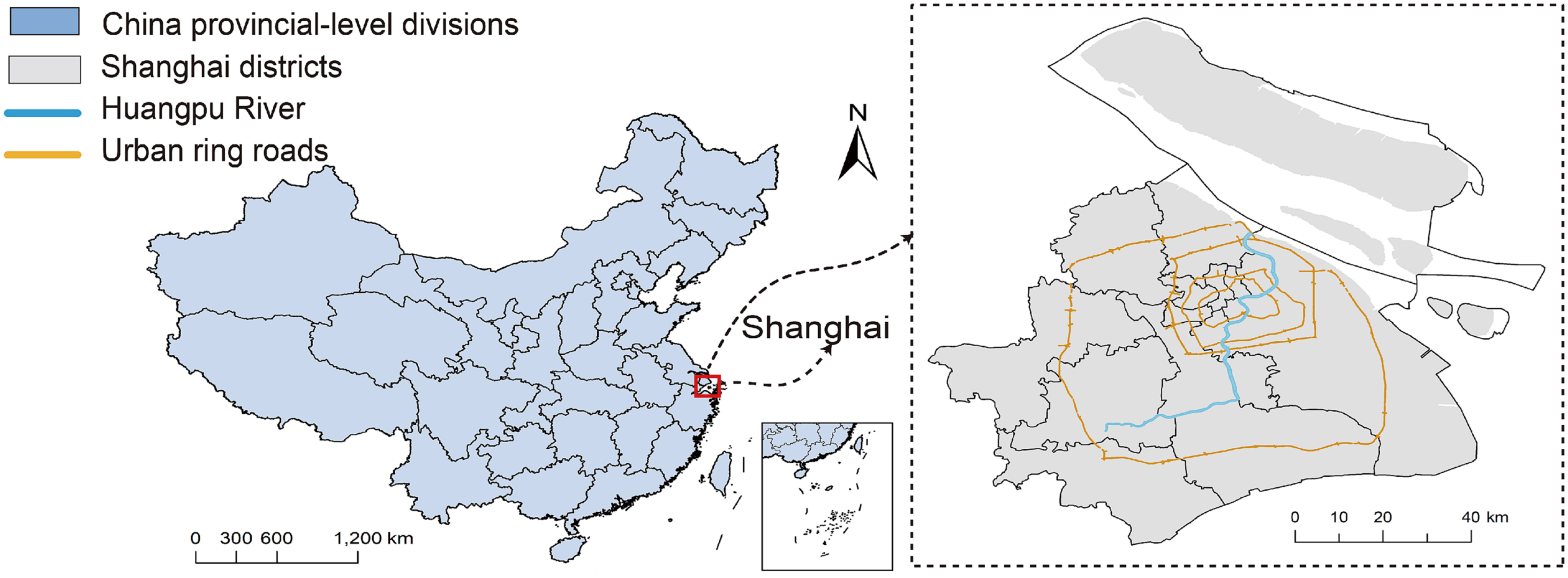
Study area.

This study selects Shanghai, China, as the empirical case to validate the proposed analytical framework (Figure 1). As a national central city and a coastal metropolis, Shanghai exhibits a polycentric spatial structure comprising a central urban core and multiple peripheral new towns. High-level urbanization has driven population distribution and activity patterns toward a state of central high-density and peripheral expansion; however, high-quality healthcare resources remain clustered in the central districts. Furthermore, Shanghai possesses a diverse and relatively comprehensive hierarchical healthcare system. The evolution of its spatial layout and operational mechanisms reflects the common challenges of healthcare supply–demand matching faced by many Chinese cities, making it a typical “strong-signal” case for diagnosing the structural frictions inherent in tiered healthcare governance.

### 2.2 Data sources

Mobile signaling data. The mobile signaling data were obtained from a major mobile network operator, comprising anonymized records for the entire month of March 2019 in Shanghai. This period was selected to capture the representative “steady state” of urban mobility, avoiding major holiday-induced fluctuations. Given the high spatial inertia of healthcare infrastructure, it serves as a robust pre-pandemic baseline for structural analysis. The dataset includes timestamps and coordinates of anonymous users at the base station level, with a temporal resolution of 1 h. To strictly adhere to data privacy and ethical standards, all data underwent irreversible anonymization and aggregation both before and after acquisition, ensuring that no personally identifiable information was included.

Hospital and vector geographic data. The directory of public general hospitals in Shanghai was sourced from the official list of medical institutions published by the Shanghai Municipal Health Commission, which includes hospital names, addresses, and grades (17–19). The final dataset comprises 242 primary care hospitals, 62 secondary hospitals, and 48 tertiary hospitals. Point-of-interest (POI) information for these hospitals was retrieved from public geographic information platforms and cross-verified with the official directory to ensure consistency in both identification and spatial positioning (20).

We designate public general hospitals as the supply units based on three primary considerations. (1) Dominance in service provision: Public general hospitals are the primary providers in the healthcare system, offering extensive coverage and representativeness. (2) Filtering behavioral noise: Our research focuses on spatial choices under general healthcare needs; excluding specialized hospitals (e.g., maternity or psychiatric facilities) helps minimize perturbations caused by specific diseases or demographic groups. (3) Core resource concentration: Public general hospitals concentrate the majority of high-quality medical resources, serving as the core objects for reflecting the supply–demand status of resource allocation and patient flow. Spatial vector data were obtained from the National Catalogue Service for Geographic Information (21).

### 2.3 Data processing and hospital-seeking trajectory identification

#### 2.3.1 Data cleaning and resident filtering

Data cleaning: Baseline data cleaning was performed on the raw signaling data, encompassing key field extraction and the handling of duplicate or anomalous records. To mitigate the false flows caused by base station handovers, consecutive records characterized by frequent switching between adjacent base stations within short intervals were merged into a single dwell point. Furthermore, anomalous data points that exceeded reasonable spatial distances within a unit of time were excluded to alleviate errors stemming from positional drift and anomalous localization (22).

Permanent resident filtering: To ensure the stability of healthcare activities during the observation period, this study focuses on the permanent population of Shanghai. Specifically, any day on which a user generated at least one valid localization record within Shanghai was marked as an “Active Day.” Only users with more than 15 active days during the observation month were retained, thereby excluding transient populations.

#### 2.3.2 Identification of residential and work locations

Based on the permanent resident filtering process, this study employs time-window constraints and the DBSCAN clustering algorithm to concurrently extract the residential and work locations of the sample users (23, 24).

Identification of residential locations. The night-time window for residential stays was defined as 22:00 to 06:00 from Monday to Sunday during the observation period. Spatiotemporal records of permanent residents within this window were extracted and subjected to spatial clustering using the DBSCAN algorithm. A cluster was identified as a stable residential location only if it satisfied the following criteria. (1) Frequency constraint: The candidate cluster must cover at least 5 nights. (2) Reliability constraint: The number of nights covered by the candidate cluster must account for more than 50% of the user’s total active nights during the entire month. When both conditions were met, the geometric center of the cluster was designated as the stable residential location, and its coordinates were recorded.

Identification of work locations. The daytime window for work-related stays was defined as 09:00–11:00 and 13:00–17:00 from Monday to Friday. Spatiotemporal records within these windows were extracted for DBSCAN clustering. A stable work location was identified based on the following criteria. (1) Frequency constraint: The candidate cluster must cover at least 5 workdays. (2) Reliability constraint: The number of workdays covered by the candidate cluster must account for more than 50% of the user’s total active workdays during the month. Upon satisfying both conditions, the geometric center of the cluster was recorded as the user’s stable work location.

#### 2.3.3 Identification of hospital-seeking trajectories

Noise elimination: To prevent long-term inpatients and hospital staff from being misidentified as healthcare-seeking visitors, samples whose residential or work coordinates fell within the hospital building footprint or its 100-meter buffer zone were excluded from the analysis.

Identification of hospital visits and destination points: For the permanent residents meeting the aforementioned criteria, their daytime activity trajectories between 08:00–11:00 and 13:00–16:00 were subjected to spatiotemporal matching with hospital locations. A valid hospital-seeking event was defined by the simultaneous fulfillment of the following heuristic criteria: (1) Spatial constraint: The dwell point must fall within the hospital building perimeter. (2) Temporal constraint: The duration of stay within the hospital area must exceed 1 h. When both conditions were satisfied, the event was recorded as a valid hospital-seeking visit, and the corresponding hospital was designated as the destination of the patient flow. The implementation of these time windows and stay thresholds aimed to filter out transient noise, such as transit passages. This study focuses specifically on outpatient-seeking behavior; consequently, night-time emergency visits and long-term hospitalizations were excluded from the research scope.

Demand unit partitioning: Using hospital locations as seeds, this study constructs Thiessen polygons to serve as demand units (25). This approach ensures spatial exclusivity and completeness while defining a nearest service area for each hospital, which serves as a geometric benchmark essential for identifying behaviors like nearest-neighbor seeking and selective bypassing. To construct a weighted, directed “supply–demand” network, a clear and consistent mapping between origins and hospitals is essential. Moreover, the “one-hospital-one-domain” setting creates a 1:1 correspondence between hospitals and demand units, enabling a precise distinction between origins and destinations within the bipartite network. This “one-hospital-one-domain” architecture is a fundamental geometric prerequisite for identifying point-to-point flow directionality. Alternative partitions, such as arbitrary grids or administrative boundaries, often result in units containing multiple hospitals or no hospitals at all, which would obscure the definition of “local” versus “bypass” behavior and hinder the precise characterization of patient choices.

**Figure 2:**
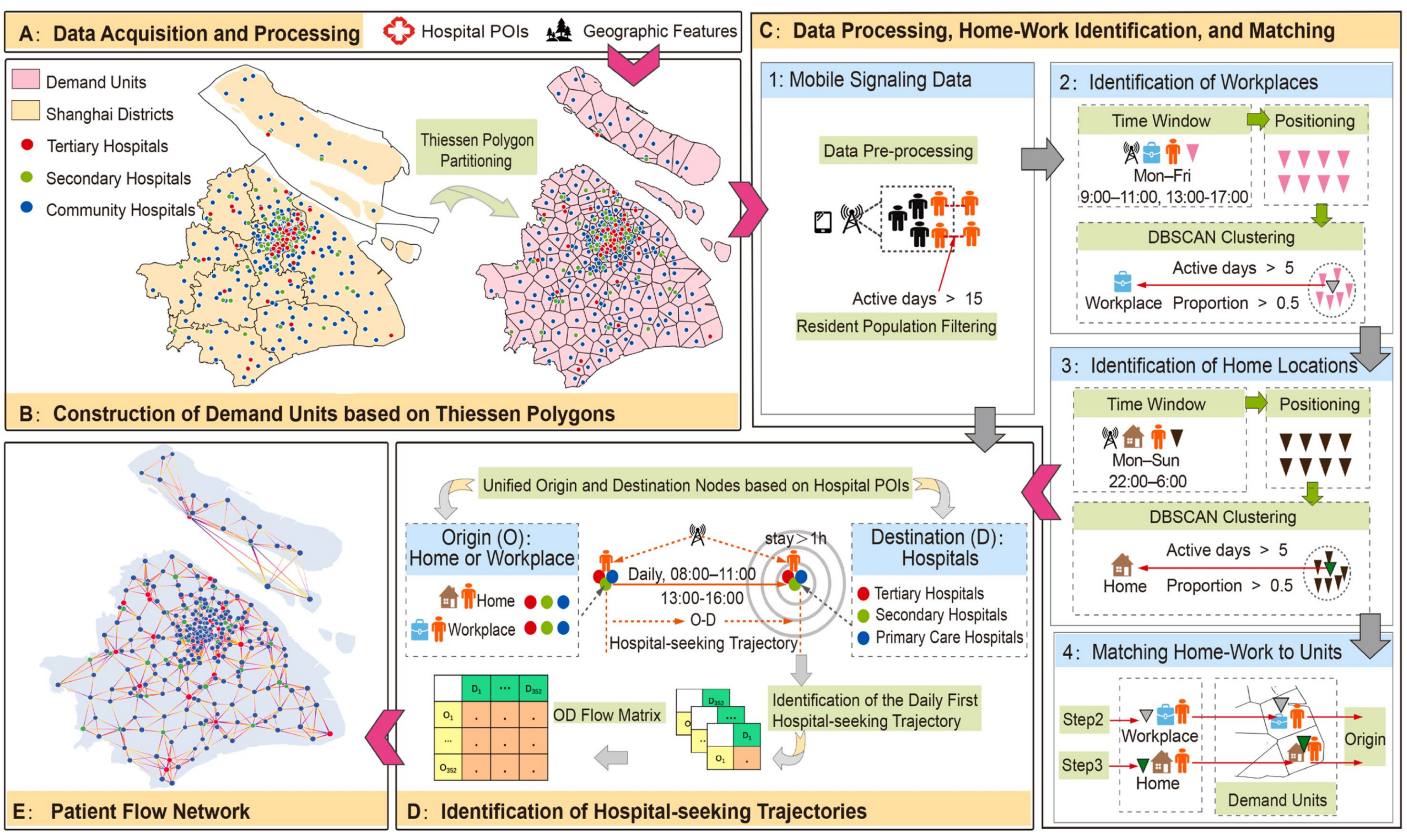
Strategic framework for the construction of the patient flow network in Shanghai. Thiessen polygons in Figure 2 represent an idealized nearest-hospital benchmark for the purpose of understanding spatial bypass behavior. These polygons are not aligned with administrative boundaries, which are shown in the background. The purpose of using Thiessen polygons is to provide a geometric reference for understanding how patients’ mobility patterns deviate from proximity-based expectations.

Identification of origins: For every valid visit, we trace the most recent workplace or residential coordinate. Through a point-in-polygon overlay, these coordinates are assigned to demand units as the path origins. To isolate initial consultation choices, only the first daily visit per user is retained as a single-hop path, excluding subsequent referrals or follow-up entries. Notably, these Thiessen polygons act merely as spatial containers for jobs-housing locations rather than imposing proximity assumptions; the actual patient flow remains entirely data-driven. Figure 2 illustrates the network construction strategy.

#### 2.3.4 Network construction and parameterization

Based on the aforementioned data processing and identification strategies, this study constructs a weighted directed patient flow network G=(O,D,E,W) , accompanied by a spatial impedance matrix R . Within this framework: The demand-side node set $\;O=\{O_{i}\}\;$ comprises Thiessen polygon demand units generated from public general hospital locations. The supply-side node set $\;D=\{D_{j}\}\;$ represents the public general hospitals. The edge set $E=\{e_{\mathrm{ij}}\}\;$ denotes the unique hospital-seeking paths from $\;O_{i}\;$ to $\;D_{j}$ . The weight matrix $\;W=\{w_{\mathrm{ij}}\}\;$ signifies the cumulative monthly patient flows from $\;O_{i}\;$ to $\;D_{j}$ for March 2019. To characterize the resistance to flow, this study develops a spatial impedance matrix $\;R=\{r_{\mathrm{ij}}\}$ . Adhering to the principle of parsimony, corrected distance is employed as a proxy for spatial impedance. The calculation involves the following stages. Monte Carlo Simulation: 2,000 sampling points are uniformly distributed within each $\;O_{i}$ . Distance averaging: The Euclidean distance from each sampling point to $\;D_{j}\;$ is calculated, with the arithmetic mean defined as the straight-line distance $\;d_{\mathrm{ij}}\;$ from $O_{i}\;$ to $\;D_{j}$ . Correction for circuitousness: To compensate for distance underestimation caused by road network detours and water barriers, a detour coefficient τ is introduced to adjust $\;d_{\mathrm{ij}}$ . Specifically, τ is set to 1.3 for standard land areas and 1.8 for cross-river paths involving Chongming Island, reflecting the significant detour costs of water crossings. Impedance definition: The final spatial impedance is defined as $\;r_{\mathrm{ij}}=d_{\mathrm{ij}}\times \tau$ , forming the matrix R .

## 3 Methods

### 3.1 Concentration measurement and null model testing

#### 3.1.1 Concentration metrics

To investigate whether the patient flow network in Shanghai exhibits a concentrated structural pattern, this study quantifies network concentration $\;M^{\mathrm{obs}}\;$ through the distributions of in-degree strength $\;S_{j}^{\text{in}}=\sum_{i}w_{\mathrm{ij}}\;$ and edge weights $\;w_{\mathrm{ij}}$ . Specifically, the Gini coefficient (26), Herfindahl–Hirschman Index ( HHI ) (27), and cumulative top-share are utilized as key statistical indicators. The cumulative top-share reflects the dominance of leading units by calculating the proportion of total network flow accounted for by the top- k ranked units. The Gini coefficient is employed to characterize the overall concentration of the distribution, where $\;x_{\left(m\right)}\;$ represents the flow values sorted in ascending order $\;(x_{\left(1\right)}\leq x_{\left(2\right)}\leq \cdots \leq x_{\left(N\right)})$ . The Gini coefficient ranges from [0,1] , with higher values indicating a more skewed or concentrated distribution. The calculation formula is as follows in Equation (1):


$$\text{Gini}=\frac{\sum_{m=1}^{N}(2m-N-1)x_{\left(m\right)}}{N\sum_{m=1}^{N}x_{\left(m\right)}}\;$$ (1)

HHI and the effective number $\;N_{\mathrm{eff}}\;$ are further employed to measure the degree of flow concentration among top-tier units. In this context, $\;x_{\left(m\right)}\;$ denotes either the in-degree strength $\;S_{m}^{\text{in}}\;$ of the m -th unit or the value of the edge weight $\;w_{\mathrm{ij}}$ , while $\;X=\sum x_{m}\;$ represents the total flow, and N signifies the total number of units. As the reciprocal of the HHI , $N_{\mathrm{eff}}\;$ provides an intuitive representation of the number of effective units that carry the bulk of the network flows. A higher HHI (corresponding to a smaller $\;N_{\mathrm{eff}}$ ) indicates that patient flow is more heavily concentrated within the leading units. The calculation formula is as follows in Equation (2):


$$\mathrm{HHI}=\sum_{m=1}^{N}{\left(\frac{x_{m}}{X}\right)}^{2},\;\;\;\;N_{\mathrm{eff}}=\frac{1}{\mathrm{HHI}}\;\;$$ (2)

#### 3.1.2 Distance decay null model

This study further constructs a null model to characterize the expected flow network under the sole constraints of demand scale and distance decay, serving as a counterfactual baseline for testing the concentration of the observed network. The underlying logic of the null model is to maintain the total outflow of each origin while redistributing the flow from $\;O_{i}\;$ to $\;D_{j}\;$ according to a power-law spatial impedance decay function. The allocation probability $\;p_{\mathrm{ij}}\;$ is defined as follows in Equation (3):


$$p_{\mathrm{ij}}=\frac{r_{\mathrm{ij}}^{-\beta }}{\sum_{u\in D}r_{\mathrm{iu}}^{-\beta }}\;\;\;\;$$ (3)

The parameter β is estimated using MLE method for multinomial distributions. While maintaining the total outflow $\;S_{i}^{\text{out}}\;$ constant, we conduct Monte Carlo simulations based on the aforementioned probabilities to generate B = 1,000 randomized networks $\;\{G^{\left(b\right)}\}\;$ which subsequently yield the simulated distribution $\{M^{\left(b\right)}\}\;$ for the corresponding statistics. To assess whether the observed statistical indicators significantly deviate from the random baseline, we employ two-tailed Monte Carlo p -values (28, 29). The two-tailed p -value is defined as twice the minimum of the left-tail and right-tail probabilities, expressed as $\;p=\min(1,2\cdot \min(p_{\text{right}},p_{\text{left}}))$ . A p -value below 0.05 indicates that the observed statistic significantly diverges from the random baseline, where the direction of this divergence is interpreted by comparing $\;M^{\mathrm{obs}}\;$ with the mean value of the null model, as calculated in Equation (4).


$$\begin{array}{l} p_{\text{right}}=\frac{1+\sum_{b=1}^{B}1(M^{\left(b\right)}\geq M^{\mathrm{obs}})}{B+1};\\ \;p_{\text{left}}=\frac{1+\sum_{b=1}^{B}1(M^{\left(b\right)}\leq M^{\mathrm{obs}})}{B+1} \end{array}$$ (4)

Given that local unit flows typically account for a significant proportion of the total volume, they may skew concentration metrics and bias the estimation of distance decay parameters. Therefore, this study utilizes two analytical scopes: The full-sample scope and the non-local scope which excludes local unit flows.

### 3.2 Channel structural decomposition and inequality

#### 3.2.1 Channel classification and structural characteristics

To disentangle the distribution and concentration characteristics of patient flows across different spatial scales, this study classifies the network edges $\;e_{\mathrm{ij}}$ into three distinct channels based on the administrative divisions and topological features of Shanghai. These categories include local unit flows, intra-district flows, and inter-district flows. Let District(·) denote the administrative division code of a node. The channel classification function for edge $\;e_{\mathrm{ij}}$ is defined as follows in Equation (5):


$$c(i,j)=\{\begin{array}{l} \text{Local}\;i=j\\ \text{Intra}\;i\neq j\;\text{and District}(O_{i})=\text{District}(D_{j})\\ \text{Inter}\;\text{District}(O_{i})\neq \text{District}(D_{j}) \end{array}\;$$ (5)

To ensure conceptual precision, we explicitly define these terms as follows. “Local unit flows” refer to patient trajectories where the origin demand unit acts as the anchor for the destination hospital (i.e., i=j ). “Intra-district flows” and “Inter-district flows” refer to non-local movements ( i≠j ) occurring within or across administrative boundaries, respectively. Based on the classification function, the global edge set E is decomposed into three subsets $\;E_{c}$ . This study calculates the number of edges $\;N_{c}=|E_{c}|$ , the total volume $\;W_{c}$ , the average edge weight $\;\bar{w}$ , and the contribution share $\;\text{Shar}e_{c}\;$ for each channel. These indicators are used to characterize the structural distribution of patient flows across different channels.

#### 3.2.2 Inequality measures of intra-channel flows

To assess whether flows within a single channel are concentrated among a few key edges, this study employs the Lorentz curve, Theil index, and effective edges for intra-group inequality measurement. The Theil index is particularly sensitive to extreme values at the tail of a distribution, making it ideal for capturing structural patterns dominated by critical edges (30). For the edge weight share $\;q_{\mathrm{ij}}=w_{\mathrm{ij}}/W_{c}\;$ within each channel, the Theil index $\;T_{c}$ and the effective edge ratio $\;\rho_{c}\;$ are calculated. A higher $\;T_{c}$ or a lower $\;\rho_{c}$ indicates that intra-channel flows are more heavily concentrated among a few primary edges. The calculation formulas are as follows in Equation (6):


$$T_{c}=\sum_{(i,j)\in E_{c}}q_{\mathrm{ij}}\ln(N_{c}q_{\mathrm{ij}}),\;\;\rho_{c}=N_{c}\cdot e^{-T_{c}}\;\;\;$$ (6)

### 3.3 Grade orientation and bypass premium

To reveal the grade orientation and spatial costs of patient flows within each channel, this study constructs indicators for the hospital-seeking upgrade rate and the bypass premium based on hospital grades and spatial impedance attributes. Given the spatial properties of Voronoi cells, local unit flows are characterized as localized visits without substantial displacement. Since these flows do not reflect grade upgrades or bypass premiums in a meaningful way, this study focuses on the $\;E_{\text{intra}}\;$ and $\;E_{\text{inter}}\;$ channels. On this basis, a hospital grade function g(·)∈{1,2,3} is established to represent primary, secondary, and tertiary hospitals, respectively. Relevant indicators are then constructed using the previously defined variables $\;w_{\mathrm{ij}}$ , $\;W_{c}$ , and $\;r_{\mathrm{ij}}$ .

#### 3.3.1 Grade share and hospital-seeking upgrade

To quantify the share of patient flows directed toward different hospital grades within channel c , this study defines the grade share $\;\pi_{c,g}$ . Furthermore, a hospital-seeking upgrade indicator variable $\;up_{\mathrm{ij}}=1[g(j)>g(i)]\;$ is defined, where g(i) denotes the grade of the generator hospital corresponding to the demand unit. Based on this indicator, the flow-weighted hospital-seeking upgrade rate $\;UR_{c}\;$ is calculated. Specifically, $\pi_{c,g}$ reflects the grade composition within a channel, while $\;UR_{c}\;$ represents the proportion of patient flows where the grade of the destination hospital exceeds that of the Voronoi generator hospital at the origin. The calculation formulas are as follows in Equation (7):


$$\pi_{c,g}=\frac{\sum_{(i,j)\in E_{c}}w_{\mathrm{ij}}1[g(j)=g]}{W_{c}},\;\;UR_{c}=\frac{\sum_{(i,j)\in E_{c}}w_{\mathrm{ij}}up_{\mathrm{ij}}}{W_{c}}\;\;\;$$ (7)

#### 3.3.2 Measurement of bypass premium

To isolate the influence of geographical location, this study employs the nearest hospital of the same grade as a benchmark to measure the bypass premium of patient flows within each channel. First, the theoretical minimum distance $\;r_{i,g}^{*}\;$ from the origin $\;O_{i}\;$ to a hospital of grade g is defined as follows in Equation (8):


$$r_{i,g}^{*}=\min\{r_{\mathrm{im}}|m\in D,g(m)=g\}\;$$ (8)

On this basis, the average bypass premium $\;BP_{c,g}\;$ for flows directed toward hospitals of grade g within channel c is defined as in Equation (9):


$$BP_{c,g}=\frac{\sum_{(i,j)\in E_{c}}w_{\mathrm{ij}}(r_{\mathrm{ij}}-r_{i,g}^{*})1[g(j)=g]}{\sum_{(i,j)\in E_{c}}w_{\mathrm{ij}}1[g(j)=g]}\;\;$$ (9)

By controlling for the target grade, this indicator characterizes the degree of bypass premium and is utilized to compare the spatial cost disparities between inter-district and intra-district channels. A positive and significant value of $\;\mathrm{\Delta B}P_{g}=BP_{\text{inter},g}-BP_{\text{intra},g}\;$ indicates a higher spatial cost for reaching the same target grade.

Crucially, we employ the nearest same-grade hospital as an operational geometric benchmark, explicitly acknowledging intra-grade heterogeneity. The bypass premium thus measures the additional spatial burden beyond this grade-conditioned minimum, rather than assuming a quality-neutral counterfactual.

#### 3.3.3 Statistical significance test for disparities

Given the long-tail distribution characteristic of patient flows, a weighted bootstrapping method is adopted to evaluate the significance of disparities in $\;\Delta \pi_{g}$ , ΔUR , and $\;\mathrm{\Delta B}P_{g}\;$ across different channels. For each channel, the edge set is resampled with replacement based on the weight $\;p_{\mathrm{ij}}=w_{\mathrm{ij}}/W_{c}$ . This procedure is repeated for B=1,000 iterations to derive the empirical distribution of the difference statistics and to construct 95% confidence intervals. If a confidence interval does not contain zero, the disparity between the channels is considered statistically significant.

## 4 Results

### 4.1 Basic network characteristics and spatial patterns

#### 4.1.1 Sparse selectivity and hierarchical concentration of patient flow

**Figure 3:**
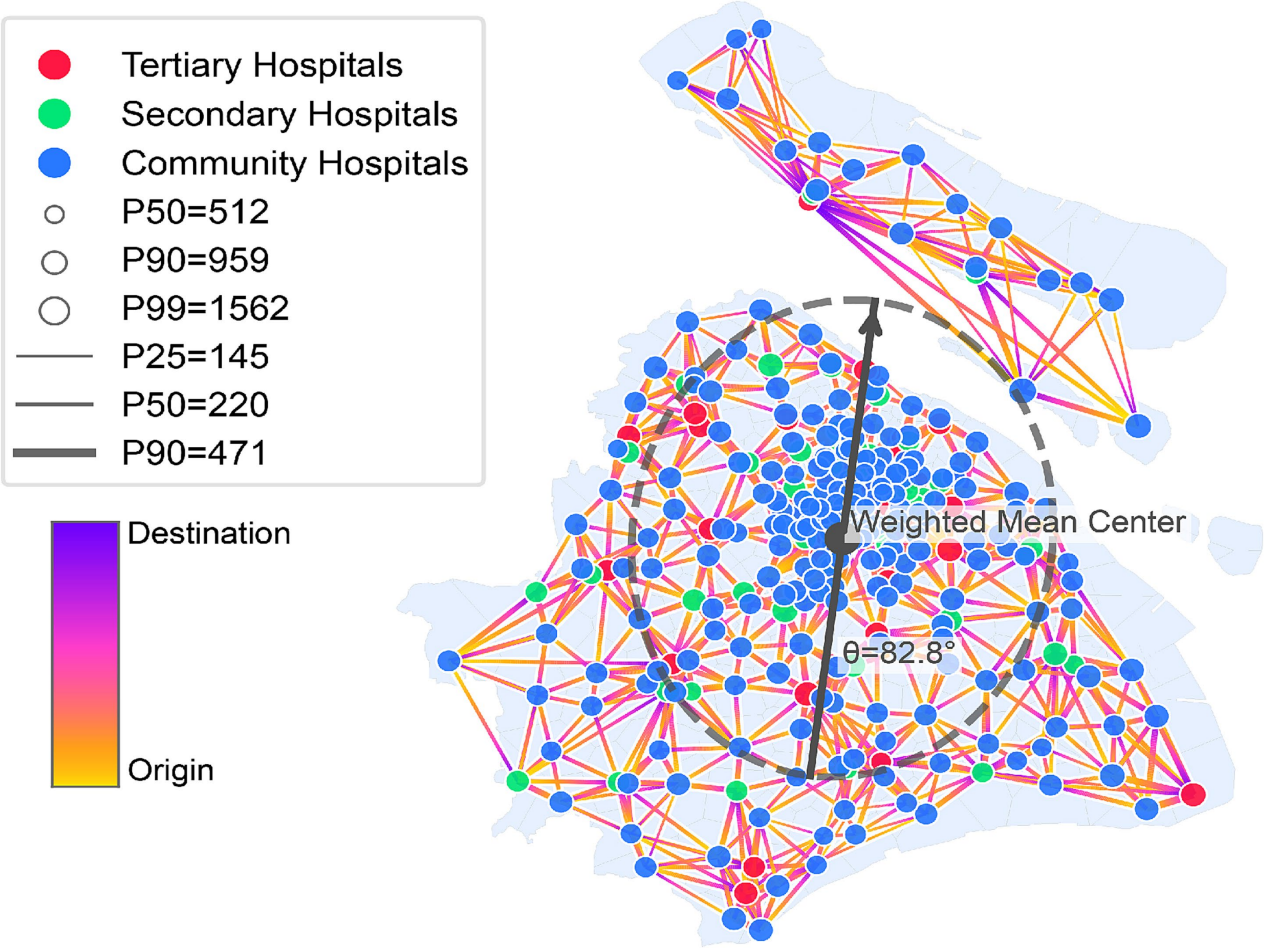
Shanghai patient flow network, March 2019 (top 5 in/outflows per node).

By leveraging mobile signaling data from March 2019, this study developed a weighted directed patient flow network for Shanghai. Drawing upon the visualization in the flow network graph shown in Figure 3 as well as the statistical syntheses in Table 1, the macro-scale patient flow across the city manifests distinct hallmarks of “sparse preferential selection” and “centripetal convergence,” accompanied by a substantial prevalence of long-distance movement.

**Table 1:** Statistical indicators of the patient flow network in Shanghai.

Dimension	Metric	Value
Network scale	Number of nodes	352
	Number of edges	17,121
	Network density	0.138
Flow composition	Total patient flow (person-times)	1,522,328
	Inflow share of top 10 hospitals	12.43%
	Share of local unit flows/Share of inter-district flows	12.55%/40.25%
	Inflow shares by hospital grade (Tertiary/Secondary/Primary)	35.70%/24.60%/39.70%
Spatial distance	Weighted average distance (km)	9.16
	Weighted distance quantiles $P_{50}/P_{90}$ (km)	6.65/18.30

The underlying network architecture is fundamentally defined by its hierarchical nature and inherent inequality. A network density of merely 0.138 underscores a patient flow network that is both sparse and characterized by a high degree of selectivity. From a capacity standpoint, tertiary hospitals shoulder 35.70% of the total patient flow, while the top-10 hospitals ranked by inflow concentrate 12.43% of the global network volume. Such figures reveal a pronounced gravitation toward head institutions, thereby consolidating a tiered utilization pattern as documented in Table 1.

#### 4.1.2 Centripetal convergence and long-distance transboundary patient flow

The observed patient flow manifests a dual trend of centripetal convergence and the transgression of administrative boundaries, which is further characterized by significant movement distances. Overall, patient flow in Shanghai exhibits multi-point clustering around high-grade hospitals, reflecting a gradual convergence from the urban periphery toward the central districts. A weighted Standard Deviational Ellipse (SDE) analysis of hospital inflows quantifies this centripetal tendency, with the resulting centroid situated in the urban center and the major axis extending along a near North–South orientation (Figure 3). This centripetal pattern is accompanied by a pronounced bypass of administrative borders and substantial travel distances. Within the total effective patient flow, the proportion of local unit flows stands at a mere 12.55%, whereas the proportion of inter-district flows reaches 40.25%. Such figures demonstrate that actual hospital-seeking behaviors have effectively surpassed the constraints of administrative boundaries. Furthermore, the weighted average distance across the network is 9.16 km, with the distance distribution showing prominent long-tail characteristics where $\;P_{50}$ = 6.65 km and $\;P_{90}$ = 18.30 km. These metrics suggest that while short-distance movement remains dominant, long-distance patient flow is a widespread phenomenon rather than an isolated occurrence (Table 1).

### 4.2 Concentration and null model testing

#### 4.2.1 Non-random aggregation characteristics based on counterfactual baselines

This study employs a null model as a stochastic benchmark to quantitatively parse the spatial organizational characteristics of actual flows by comparing the structural features of the full sample with those of the sample excluding local unit flows. Results in Table 2 indicate that the distance decay parameter β for the sample excluding local unit flows is 2.44, which is higher than the β = 2.07 observed for the full sample. Such a disparity suggests that cross-unit movements are more sensitive to spatial distance.

**Table 2:** Summary of network concentration and edge structural metrics.

Dimension	Metric	Full			Off-diagonal (non-local)		
		Observed	Null mean	Obs/Null	Observed	Null mean	Obs/Null
Distance decay	β	2.07	–	–	2.44	–	–
Node level	Gini	0.410	0.180	2.28↑^**^	0.468	0.222	2.11↑^**^
	$N_{\mathrm{eff}}$	196.20	319.60	0.61↓^**^	171.70	305.10	0.56↓^**^
	Top-10 share	12.40%	4.30%	2.88↑^**^	14.00%	4.80%	2.92↑^**^
Edge level	Edges	17,121	25,074	0.68↓^**^	16,769	21,657	0.77↓^**^
	Gini	0.592	0.640	0.92↓^**^	0.565	0.611	0.92↓^**^
	$N_{\mathrm{eff}}$	5,233	3,100	1.69↑^**^	5,907	4,726	1.25↑^**^
	Top 1% share	10.10%	23.20%	0.44↓^**^	8.70%	14.10%	0.62↓^**^

^*^p < 0.05, ^**^p < 0.01, ^***^p < 0.001. The contrast between node and edge levels reveals a dual structural pattern. Node-level metrics indicate stronger than null inflow concentration (higher Gini, lower $N_{\mathrm{eff}}$ ), while edge-level metrics indicate a more diffusive distribution across routes (higher $N_{\mathrm{eff}}$ , lower Top-1% share). Together, this suggests concentration in destinations rather than domination by a few extreme routes.

The distribution of hospital inflows exhibits significant concentration, with the observed Gini coefficient of 0.410 being considerably higher than the 0.180 recorded for the null model. Furthermore, the number of effective hospitals across the network is approximately 196, representing only 61.39% of the value expected under the null model. The top 10 hospitals carry 12.40% of the total patient flow, a figure that is 2.88 times higher than the stochastic expectation. These metrics collectively demonstrate that regardless of whether local unit flows are excluded, actual patient flow remains highly sensitive to spatial distance and consistently exhibits a degree of concentration far exceeding random probability at the level of hospital inflows.

#### 4.2.2 Counterfactual characteristics of flow distribution: sparse paths and spatial diffusion

The distribution of edges is characterized by numerical sparsity and discrete patterns. Regarding the numerical scale, the observed network contains 17,110 edges, yielding an observation-to-null model ratio of 0.68, which indicates that the edge density of the observed network is significantly lower than stochastic expectations. At the distributional level, the Gini coefficient for edge-level patient flow in the observed network is 0.592, which is notably lower than the 0.640 recorded for the null model. Furthermore, the effective number of edges ( $N_{\mathrm{eff}}$ ) in the observed network significantly exceeds the random expectation (Table 2). These results suggest that compared to the null model, which excessively accumulates flow on short-distance edges, the observed patient flows exhibit greater spillover and diffusion across different directions, resulting in a relatively more even distribution.

Detailed analysis further reveals that the flow share of the top 1% of leading edges accounts for only 43.53% of the null model’s value (Table 2). These results suggest that whereas the null model tends to excessively concentrate volume on short-distance edges, the actual patient flow manifests a pattern of greater overflow and diffusion across diverse directions, resulting in a relatively more balanced distribution.

Taken together with the node-level results, the network exhibits a concentrated destination structure where hospital inflows are more unequal than the null, but a relatively diffusive route structure where flows are distributed across more effective edges than the null.

### 4.3 Channel structure decomposition and internal inequality

#### 4.3.1 Imbalanced distribution of edge scales and flow intensities within each channel

A significant divergence exists between the topological structure and functional carrying capacity within the patient flow network: While inter-district channels underpin the broad-area connectivity of the network, the local unit and intra-district channels constitute the functional core that shoulders the majority of actual patient flow.

**Figure 4:**
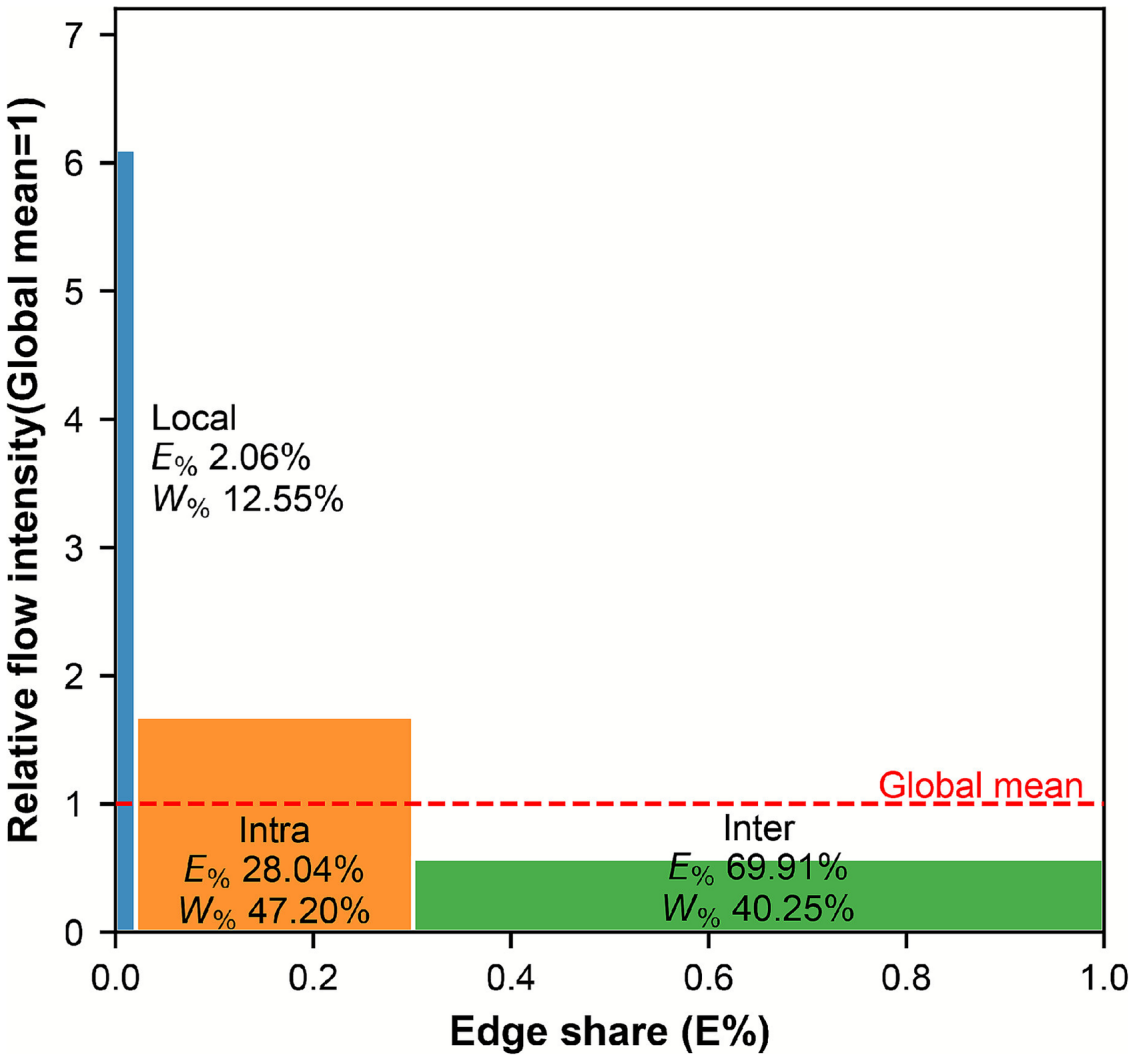
Distribution of edge share and flow concentration within different patient flow channels.

The local unit flow channel is characterized by low connectivity but high-intensity aggregation. Although the number of edges in this channel accounts for only 2.06% of the global network, it carries 12.55% of the total patient flow (Figure 4). As indicated in Table 3, the average edge weight within this channel reaches 542.78 person-times, which is 6.10 times the global average. This disparity reflects the exceptionally high frequency of local hospital-seeking occurrences and the superior utilization efficiency of these local channels.

**Table 3:** Structural contribution and inequality metrics of patient flow channels.

Channel	Edges	Edge share	Flows	Flow share	Mean flow	Theil	Effective size	Effective ratio
	$E_{C}$	$E_{\%}$	$W_{C}$	$W_{\%}$	${\bar{w}}_{C}$	$T_{C}$	$\rho_{c}$	$\rho_{c}/E_{C}$
Local	352	2.06%	191,058	12.55%	542.78	0.187	291.93	0.829
Intra	4,800	28.04%	718,553	47.20%	149.70	0.436	3,103.83	0.647
Inter	11,969	69.91%	612,717	40.25%	51.19	0.453	7,612.17	0.636
Global	17,121	100.00%	1,522,328	100.00%	88.92	–	–	–

The intra-district channel carries nearly half of the global volume, thereby establishing its predominance in terms of flow bearing capacity. While the number of edges in this channel accounts for 28.04% of the network total, it supports 47.20% of the actual patient flow, making it the most significant channel by flow share. This indicates that cross-hospital movement within administrative districts represents the primary mode of patient flow (Figure 4).

In contrast, the inter-district channel is characterized by low density and extensive spatial coverage. This channel accounts for a substantial 69.91% of the total edge scale, providing the foundational connectivity for regional accessibility. However, it carries only 40.25% of the total patient flow volume (Table 3). Furthermore, its average edge weight stands at 51.19 person-times, which is merely 0.57 times the global average. The observed inverse relationship between edge share and flow share suggests that although inter-district patient flow benefits from the most extensive selection of paths, the average carrying capacity of individual paths remains relatively weak.

#### 4.3.2 Gradient heterogeneity of flow distribution within each channel

**Figure 5:**
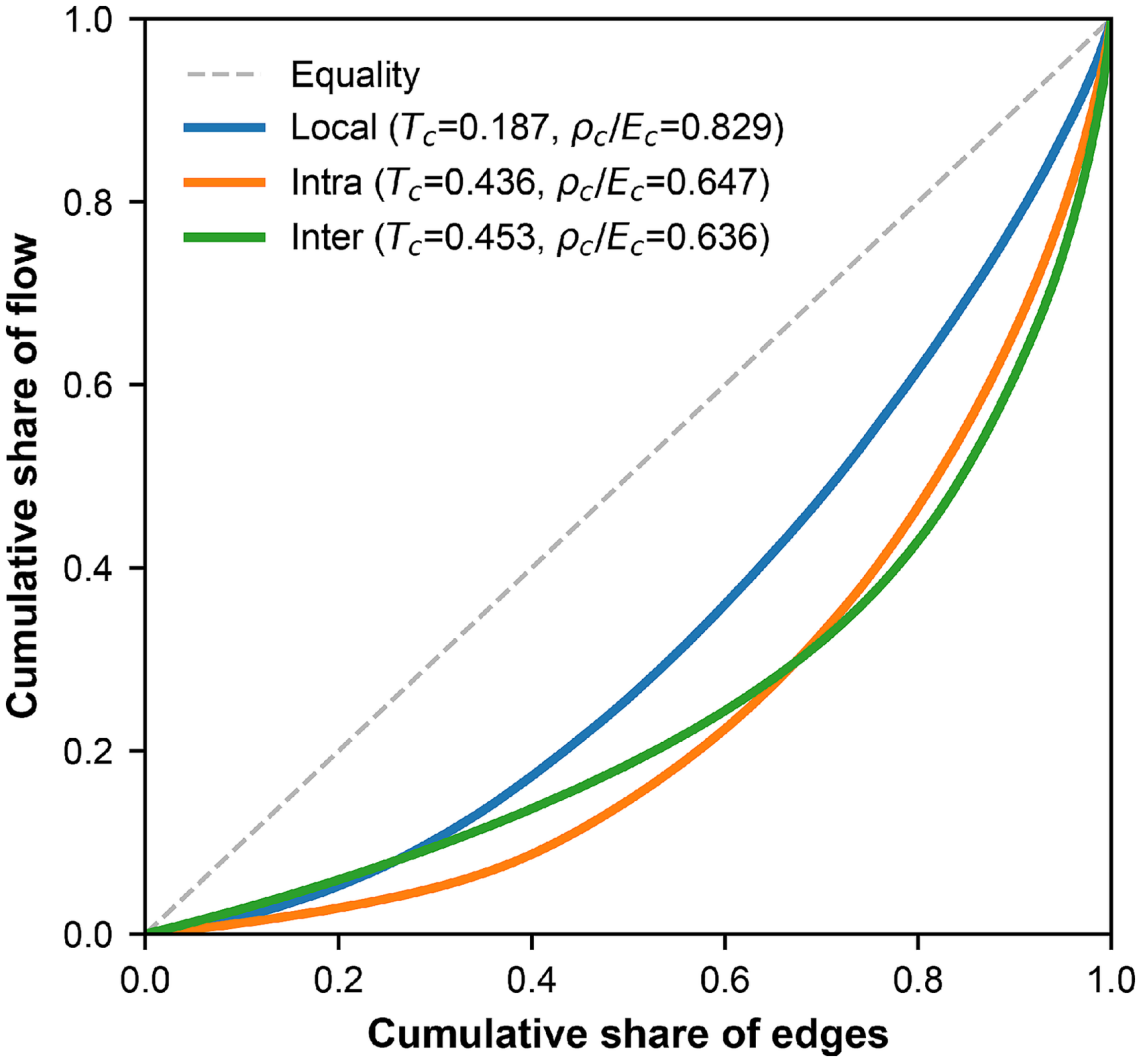
Lorenz curves of patient flow distribution within each channel.

Lorenz curves (Figure 5) and Table 3 reveal a gradient heterogeneity in patient flow distribution, where expanding spatial scales trigger a shift from local uniformity toward inter-district concentration.

The local unit flow exhibits universal and homogeneous accessibility. Its Lorenz curve remains nearest the diagonal, yielding a Theil index of 0.187 and an effective edge ratio of 0.829. These metrics indicate that volume within demand units is not excessively concentrated, affirming the spatial equity of primary care hospital services as a fundamental safety net.

The intra-district channel is characterized by transitional concentration. When the scope of patient flows expands from demand units to the entire administrative district, the Theil index rises to 0.436 while the effective ratio drops to 0.647 as shown in Table 3. These findings indicate that once patient flows breach the boundaries of individual demand units, the distribution of volume is no longer uniform, demonstrating a clear orientation in healthcare seeking behavior.

The inter-district channel exhibits a state of extreme structural polarization. While the preceding analysis indicates that the average edge flow of inter-district pathways is relatively low, this average value obscures a profound internal structural differentiation. This category of pathways possesses the highest Theil index at 0.453 and the lowest effective edge ratio at 0.636 as detailed in Table 3. As illustrated by the Lorenz curve in Figure 5, the significant deviation from the diagonal line confirms a severe head effect within the network structure. This pattern demonstrates that the vast majority of inter-district edges carry only negligible long-tail flows, whereas the primary volume of patient flows is highly concentrated on a limited number of critical backbone pathways.

### 4.4 Grade orientation and bypass premium

#### 4.4.1 Differentiation of flow structures: upgrading rate and functional neutrality

**Figure 6:**
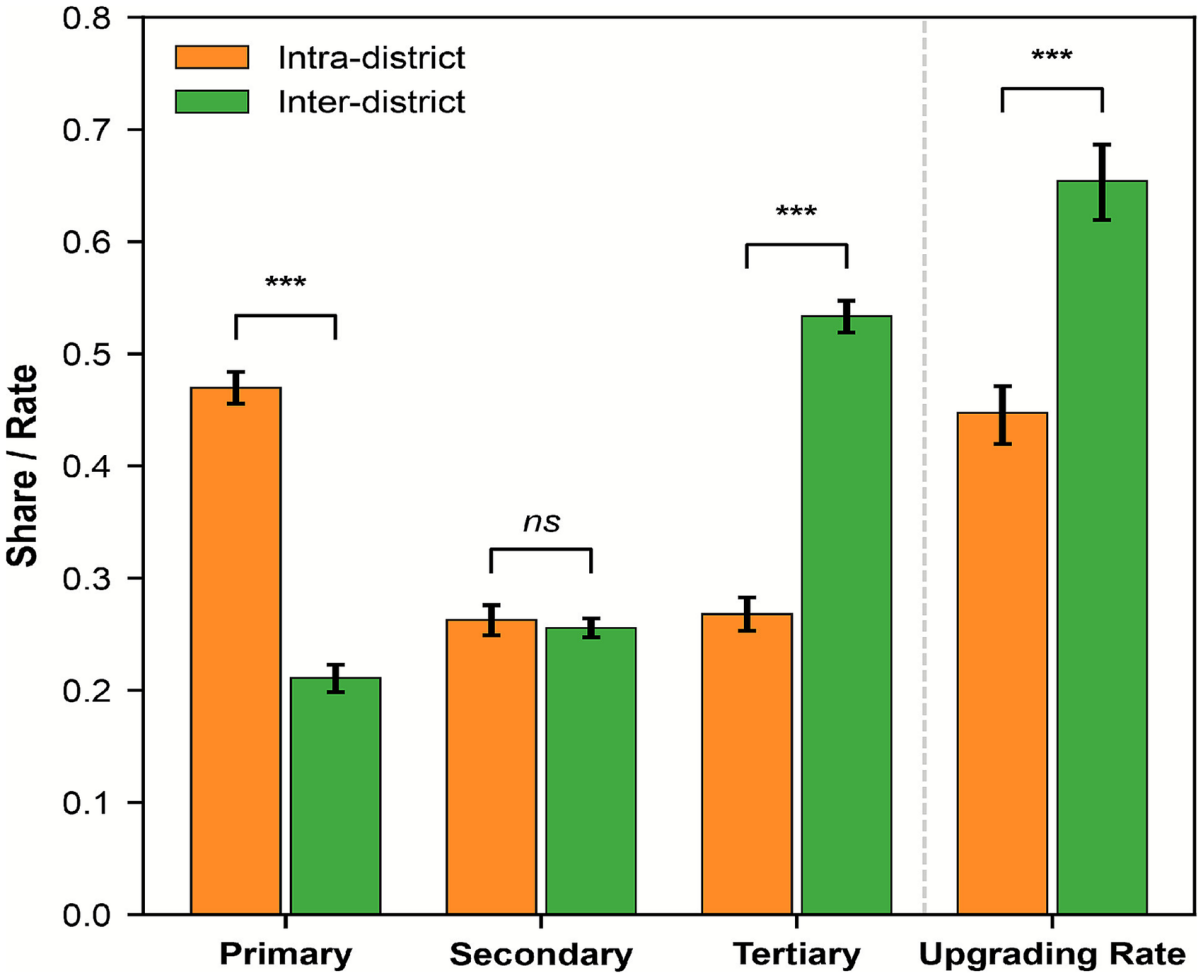
Decomposition of channel structures and medical upgrading rates.

The patient flow within intra-district channels manifests a hierarchical distribution pattern characterized by a predominance of primary-level services. As illustrated in Figure 6 and Table 4, primary care hospitals within these channels accommodate 47% of the total volume, while secondary and tertiary hospitals account for 26.20 and 26.80%, respectively. These results indicate that within the various administrative districts of Shanghai, patient flow adheres to a conventional progression from the primary level toward higher-grade facilities.

**Table 4:** Flow structure, upgrading tendency, and bypass premium by channel type.

Dimension	Metric	Intra-district	Inter-district	Differential effect	95% Confidence interval
Flow structure	Primary care hospital	0.470	0.211	−0.259^***^	[−0.278, −0.242]
	Secondary hospital	0.262	0.256	−0.007	[−0.022, 0.009]
	Tertiary hospital	0.268	0.534	0.266^***^	[0.247, 0.284]
Upgrading tendency	Upgrading rate	0.447	0.654	0.207^***^	[0.181, 0.232]
Bypass premium	Secondary hospital	2.553	7.525	4.968^***^	[4.318, 5.625]
	Tertiary hospital	2.238	10.237	7.995^***^	[7.249, 8.794]

^*^p < 0.05, ^**^p < 0.01, ^***^p < 0.001.

Inter-district patient flows exhibit a pronounced shift toward higher-grade facilities. Compared to intra-district flows, the share of primary care hospitals drops to 21.10% while tertiary hospitals surge to 53.40%, becoming the dominant destination. Moreover, the hospital-seeking upgrade rate reaches 65.40% in inter-district channels, significantly exceeding the 44.70% found in intra-district channels. These disparities, except for the secondary hospital proportion, remain statistically significant under Bootstrap testing (Table 4).

Intriguingly, secondary hospitals manifest what we term “functional neutrality.” Figure 6 and Table 4 show no significant difference in their share between intra-district (26.20%) and inter-district (25.60%) channels. It suggests that despite increased physical distances, the market share of secondary hospitals remains structurally stable. Such a neutral response to administrative boundaries indicates that their flow distribution possesses high spatial-structural stability. This represents an outcome-level structural observation rather than direct evidence of institutional performance.

#### 4.4.2 Deconstruction of spatial costs: active selection premium mechanisms

**Figure 7:**
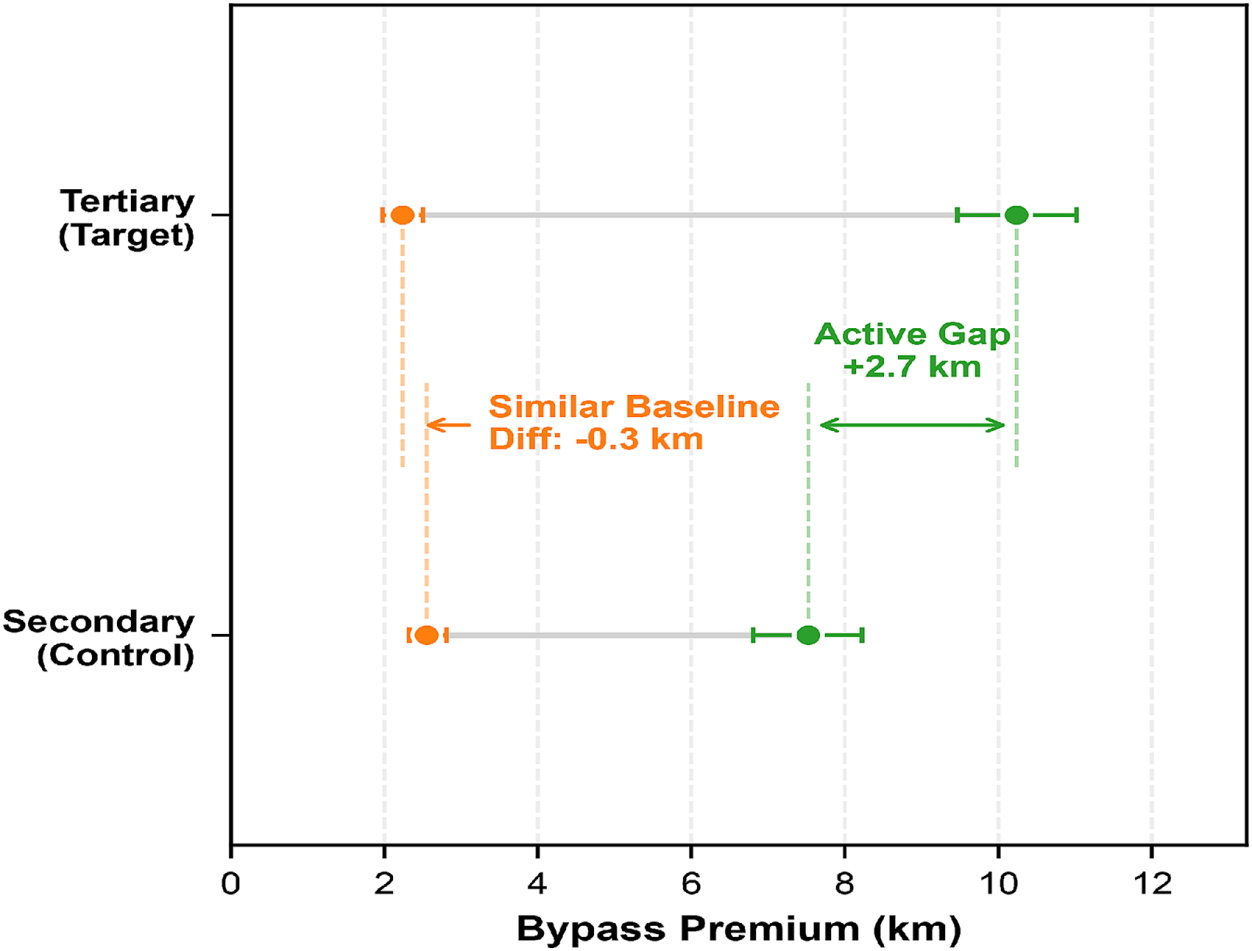
Decomposition of bypass premium and the active selection gap.

Table 4 and Figure 7 show that intra-district bypass premiums for secondary and tertiary hospitals remain closely aligned at 2.55 km and 2.24 km, respectively. This suggests that patient flows within these channels are largely driven by proximity, and grade related differences are not associated with a clear additional distance burden. In contrast, the bypass premium in inter-district channels surges, reflecting the substantial spatial impedance of cross-district healthcare seeking.

Secondary hospital metrics establish the pragmatic baseline for inter-district flows: Their bypass premium increases from 2.55 km in intra-district channels to 7.53 km in inter-district channels. Given the functional neutrality of secondary hospital flows, we interpret this 7.53 km value as a proxy for the baseline structural distance burden under grade conditioning and the observed channel structure. We refer to this component as passive structural friction rather than pure institutional friction.

Tertiary hospitals exhibit a pronounced active selection premium, with their bypass premium escalating to 10.24 km. Using the 7.53 km secondary hospital baseline as a reference, the remaining 2.71 km represents an additional premium beyond the baseline (Figure 7).

## 5 Discussion

### 5.1 Erosion of geographical proximity rules: non-random polarization and supply–demand mismatch

Existing studies typically operate under the geographical proximity rule, assuming that patients prioritize distance as the primary cost and prefer the nearest available hospital (31). However, the null model tests in this study challenge this premise, revealing a distinct non-random structural polarization in hierarchical service usage. Specifically, patient flow concentration significantly exceeds random expectations, showing a pronounced aggregation toward top-tier hospitals. Conversely, edge concentration falls below random expectations, manifesting a counter-intuitive pattern of spatial diffusion. These findings suggest that under modern efficient transportation networks, spatial impedance is being fundamentally reconstructed by patients’ rigid preferences for high-quality services. This further validates that the systemic imbalance within the current hierarchical healthcare system stems not merely from insufficient spatial coverage but from excessive vertical disparities in quality. Consequently, strategies relying solely on increasing facility quantity and coverage while neglecting quality homogenization will fail to mitigate this systemic imbalance driven by quality preferences (32, 33).

### 5.2 Reshaping boundary perceptions: layering mechanisms of healthcare under administrative fragmentation

Unlike previous studies that view administrative boundaries merely as rigid barriers or service endpoints (34), our channel analysis reveals that they function as a selective value filter. While cross-district flows offer broad connectivity, actual patient flows do not spread randomly but are intensely locked into backbone routes leading exclusively to tertiary hospitals. This suggests that the administrative boundary acts like a sieve that effectively retains routine healthcare needs within local districts, aligning with the policy goal of strengthening primary care hospitals, while remaining permeable to high-value demands where patients seek top-tier resources. This filtering effect stems from a rational cost–benefit trade-off where the passive structural friction of approximately 7.53 km serves as a threshold high enough to discourage cross-district travel for common ailments but is readily overcome by patients with critical conditions who prioritize quality over proximity. Consequently, inter-district flow represents a targeted and efficient resource-matching process rather than disorderly spatial sprawl, a finding that resonates with regional governance theories on territorial efficiency (35). However, this efficiency acts as a double-edged sword because the boundary mechanism enforces a necessary functional division but simultaneously solidifies the cost barrier, deepening the spatial inequity of access between the resource-rich city center and the periphery.

### 5.3 Cost deconstruction: passive structural friction and selection premium in inter-district flows

Addressing the challenge of disentangling rigid spatial constraints from active quality seeking remains difficult in the analysis of long-distance patient flow. This study moves beyond treating these factors as a black box by using the bypass premium metric to provide an outcome-level structural accounting of travel costs under grade conditioning. By establishing the observed neutrality pattern of secondary hospitals as a pragmatic physical baseline, we structurally decompose the 10.24 km bypass premium observed for tertiary hospitals.

Data indicates that 73.54% (7.53 km) of this distance corresponds to the baseline component under the grade conditioned benchmark and the observed channel structure. Conversely, the remaining 26.46% (2.71 km) can be interpreted as the net active selection premium, reflecting additional distance burdens beyond the baseline that are consistent with patients incurring extra travel to access tertiary care. Importantly, this decomposition is intended as an outcome-level structural accounting under grade conditioning, rather than definitive causal attribution. This separation complements traditional accessibility models by quantifying the baseline burden versus the residual premium in interdistrict flows, suggesting that substantial additional distance burdens persist even after baseline impedance is accounted for (36).

### 5.4 Middle tier weakening: functional neutrality of secondary hospitals

Our analysis identifies a structural pattern within the secondary hospital tier that we characterize as “middle tier weakening.” Secondary hospitals maintain a relatively stable market share of approximately 26% across both intra-district and inter-district channels. This stability represents an outcome level observation rather than direct evidence of institutional failure; within our flow-based framework, it indicates “functional neutrality,” namely limited differentiation of the secondary tier across spatial channels.

This phenomenon reflects a “sandwich” dilemma: Community health centers capture routine local demand through proximity, while tertiary hospitals attract longer distance flows due to perceived capability and specialization. We acknowledge that multiple conflated mechanisms likely contribute to this pattern, including capacity constraints, specialty mismatches, policy induced referral pathways, and patient awareness, which cannot be fully disentangled using mobility data alone. Nevertheless, the observed polarization implies that strengthening the functional positioning of secondary hospitals, such as through clearer specialization and technical decentralization, is crucial for improving hierarchical coordination beyond administrative referral mandates alone (37, 38).

### 5.5 Limitations and future research directions

This study utilizes Shanghai as a strong-signal case of a hierarchical healthcare system to demonstrate our proposed dynamic diagnostic framework. While the specific empirical findings, such as the pronounced filtering effect of administrative boundaries, are deeply rooted in Shanghai’s institutional context, the analytical constructs proposed herein possess broader applicability. Specifically, the channel decomposition method and the bypass premium index function as generalizable diagnostic tools designed to quantify the intensity of spatial and institutional friction. In cities with weaker administrative control, the same framework would likely reveal distinct structural patterns (e.g., higher cross-boundary permeability), thereby serving as a standardized metric for future comparative research.

Notwithstanding the transferability of the framework, specific constraints regarding data attributes and model assumptions remain and warrant future improvement.

1. The lack of diagnostic information limits the granularity of behavioral analysis. While mobile signaling data offers high spatiotemporal resolution, it inherently lacks clinical context, such as specific disease types or medical expenses. Since the motivation for cross-district mobility varies significantly between intractable diseases and common ailments, this data limitation makes it difficult to distinguish the heterogeneous contributions of different conditions to the “bypass premium.” This may result in a somewhat homogenized understanding of the healthcare needs of diverse populations.
2. The identification of hospital-seeking trajectories relies on spatiotemporal heuristic criteria rather than clinical records. Although this identification method is statistically significant at the aggregate level, it cannot fully filter out “pseudo-patient flows,” such as accompanying family members or non-medical visitors, at the individual level. Additionally, as this study focuses specifically on outpatient behavior, excluding nighttime emergencies and long-term inpatients, it may partially underestimate the actual peak load of the urban healthcare system.
3. Uncertainties exist in spatial unit partitioning and impedance measurement. The use of Thiessen polygons for demand unit partitioning ensures spatial continuity but may be subject to the Modifiable Areal Unit Problem (MAUP), potentially influencing structural results. Furthermore, the spatial impedance metric, based on corrected distance, serves as a static simplification. It fails to capture dynamic traffic factors, such as peak congestion and public transit transfers, potentially biasing the estimation of real-world time costs.
4. Future research should prioritize a “multi-source data fusion” paradigm. With the advancement of digital public health, researchers should explore secure linkages between mobile mobility data, medical insurance records, and Electronic Health Records (EHR) under privacy-preserving frameworks. Such integration would validate the accuracy of trajectory identification and enable a more precise model that unifies trajectory tracking with disease identification and cost attribution. Crucially, this paradigm will allow researchers to disentangle the complex mechanisms behind the observed functional neutrality of the middle tier. By accounting for insurance incentives and clinical diagnostic records, future studies can clarify whether the “sandwich dilemma” of secondary hospitals stems from service specialization mismatches or policy-induced constraints. For instance, spatial regression models incorporating insurance reimbursement differentials could isolate the impact of financial incentives on patient awareness and hospital choice. Ultimately, this approach will provide robust micro-level evidence for the spatial governance of resource allocation and the optimization of the hierarchical healthcare system.

## 6 Conclusion and recommendations

### 6.1 Conclusion

Taking Shanghai as a representative city case study, this research utilizes mobile signaling data to construct a comprehensive patient flow network, thereby providing a quantitative framework to analyze non-random structural polarization, micro-behavioral patterns, and the deconstruction of healthcare-seeking costs through null model tests, channel decomposition, and bypass premium measurements.

The primary conclusions are as follows. First, patient flow in Shanghai exhibits dual characteristics of wide-area diffusion and centripetal convergence, where rigid demand for high-quality resources overrides geographical proximity constraints and leads to hospital aggregation far exceeding random expectations. Second, administrative boundaries act as a selective value filter, causing inter-district flows to polarize along backbone paths to tertiary hospitals, which facilitates hierarchical order but solidifies spatial inequality. Third, regarding the inter-district bypass premium, 73.54% stems from passive structural friction, while 26.46% represents the net selection premium actively paid by residents for superior resources. Fourth, secondary hospitals manifest functional neutrality, experiencing a structural vacancy where they fail to serve as pivotal hubs within the hierarchical medical system.

### 6.2 Policy recommendations

The first priority is mitigating the passive structural friction that hinders equitable access. Transport authorities should establish dedicated medical shuttle networks linking suburban towns to tertiary centers to fill existing transit gaps. Access should be restricted via a referral-based pass system, ensuring these resources prioritize complex cases and transforming rigid boundaries into managed screening channels.

Financial levers are required to guide the active selection behavior driven by quality preferences. Widening reimbursement gaps and increasing co-payment ratios for non-emergency patients who bypass referrals will internalize the social costs of congestion. This mechanism effectively redirects common ailments to local facilities while preserving tertiary capacity for critical medical needs.

Resolving the functional neutrality of secondary hospitals requires a shift from comprehensive competition to specialized differentiation. By pivoting toward rehabilitation or chronic care and deploying expert teams through medical consortiums, these institutions can establish district-level brand disciplines. This allows secondary hospitals to effectively intercept referrals and restore their role as pivotal hubs.

Finally, healthcare governance must evolve toward dynamic resource allocation informed by real-world mobility. Health commissions should utilize healthcare flow heatmaps to identify service deficits and adjust resource quotas, such as beds and subsidies, based on actual cross-district loads. This ensures the institutional structure adapts to the fluid reality of patient mobility rather than static administrative headcounts.

## Data Availability

The original contributions presented in the study are included in the article/supplementary material, further inquiries can be directed to the corresponding author.

## References

[1] Wang YF Zhai DQ Xie WQ Huang S. Spatial optimization of hierarchical healthcare facilities driven by multi-source data: a case study of Shenyang, China. Front Public Health. (2025) 13:22. doi: 10.3389/fpubh.2025.1640070, 40786168 PMC12331506

[2] Gong SZ Gao Y Zhang F Mu L Kang CG Liu Y. Evaluating healthcare resource inequality in Beijing, China based on an improved spatial accessibility measurement. Trans GIS. (2021) 25:1504–21. doi: 10.1111/tgis.12737

[3] Gan ZX Li LC Miao HZ Zhao RJ Yang M. Can public transportation improve equity in high-level healthcare time accessibility? Transport Res Part D-Transport Environ. (2025) 145:19. doi: 10.1016/j.trd.2025.104804

[4] Du FY Wang JE. Towards optimizing healthcare Facility in Urban Space: addressing diurnal population shifts. J Geogr Sci. (2025) 35:2490–508. doi: 10.1007/s11442-025-2422-4

[5] Lendado TA Bitew S Elias F Samuel S Assele DD Asefa M. Effect of hospital attributes on patient preference among outpatient attendants in Wolaita zone, southern Ethiopia: discrete choice experiment study. BMC Health Serv Res. (2022) 22:11. doi: 10.1186/s12913-022-07874-x, 35581592 PMC9110630

[6] Du F Wang J Mao L Liu Y. Spatial equity in healthcare access: an opportunity-utilization perspective. Cities. (2024) 155:105424. doi: 10.1016/j.cities.2024.105424

[7] Zhang T Xu YJ Ren JP Sun LQ Liu CJ. Inequality in the distribution of health resources and health Services in China: hospitals versus primary care institutions. Int J Equity Health. (2017) 16:8. doi: 10.1186/s12939-017-0543-9, 28253876 PMC5335774

[8] Chen BQ Jin FL. Spatial distribution, regional differences, and dynamic evolution of the medical and health services supply in China. Front Public Health. (2022) 10:20. doi: 10.3389/fpubh.2022.1020402, 36211684 PMC9540227

[9] Cheng G Zeng XK Duan L Lu XP Sun HC Jiang T . Spatial difference analysis for accessibility to high level hospitals based on travel time in Shenzhen, China. abitat Int. (2016) 53:485–94. doi: 10.1016/j.habitatint.2015.12.023

[10] Chen PF Jian IY Zhang L Siu KWM Liu JX Liu ZW . Towards a smart healthy city: a generalised flow-based 2sfca method for incorporating actual mobility data in healthcare accessibility evaluation. Travel Behav Soc. (2024) 34:13. doi: 10.1016/j.tbs.2023.100706

[11] Nie Q Wu JJ Yan XY Liu JH Wang J. Human migration patterns in China with the resume data. Mod Phys Lett B. (2019) 33:14. doi: 10.1142/s0217984919500295

[12] Simini F González MC Maritan A Barabási AL. A universal model for mobility and migration patterns. Nature. (2012) 484:96–100. doi: 10.1038/nature10856, 22367540

[13] Xiang B Hong M Guo F Wei W. Spatial structure and mechanism of Cross-City patient mobility network in the Yangtze River Economic Belt of China. J Urban Manag. (2025) 14:562–76. doi: 10.1016/j.jum.2024.11.013

[14] Li PR Zhang HR Li WJ Huang D Guo ZL Chen JY . Geoavatar: a big Mobile phone positioning data-driven method for individualized Pseudo personal mobility data generation. Comput Environ Urban Syst. (2025) 119:12. doi: 10.1016/j.compenvurbsys.2025.102252

[15] An C O'Malley AJ Rockmore DN Stock CD. Analysis of the U.S. patient referral network. Stat Med. (2018) 37:847–66. doi: 10.1002/sim.7565, 29205445 PMC5799011

[16] Caglar Koylu SD Guo D Celik RN. Analysis of big patient mobility data for identifying medical regions, Spatio-temporal characteristics and care demands of patients on the move. Int J Health Geogr. (2018) 17:32. doi: 10.1186/s12942-018-0152-x, 30071864 PMC6071389

[17] Shanghai Municipal Health Commission. Directory of Shanghai Community Health Service Centers (2019). Available online at: https://wsjkw.sh.gov.cn/fwjg/20180601/0012-55892.html (Accessed July 21, 2020).

[18] Shanghai Municipal Health Commission. Directory of Secondary Healthcare Institutions in Shanghai (2019). Available online at: https://wsjkw.sh.gov.cn/fwjg/20180815/0012-61976.html (Accessed July 21, 2020).

[19] Shanghai Municipal Health Commission. Directory of Tertiary Healthcare Institutions in Shanghai (2019). Available online at: https://wsjkw.sh.gov.cn/fwjg/20180601/0012-55891.html (Accessed July 21, 2020).

[20] Amap. Point-of-Interest (Poi) Data of Healthcare Facilities in Shanghai (2019). Available online at: https://lbs.amap.com/ (Accessed March 12, 2020).

[21] National Catalogue Service for Geographic Information. National Fundamental Geographic Information Database (Shanghai Section) (2019). Available online at: https://www.webmap.cn/ (Accessed March 12, 2020).

[22] Widhalm P Yang YX Ulm M Athavale S González MC. Discovering urban activity patterns in cell phone data. Transportation. (2015) 42:597–623. doi: 10.1007/s11116-015-9598-x

[23] Birant D Kut A. St-Dbscan: an algorithm for clustering spatial-temp Oral data. Data Knowl Eng. (2007) 60:208–21. doi: 10.1016/j.datak.2006.01.013

[24] Zheng Y. Trajectory data mining: an overview. ACM Trans Intell Syst Technol. (2015) 6:41. doi: 10.1145/2743025

[25] Barbosa H Barthelemy M Ghoshal G James CR Lenormand M Louail T . Human mobility: models and applications. Phys Rep. (2018) 734:1–74. doi: 10.1016/j.physrep.2018.01.001

[26] Gini C. Measurement of inequality of incomes. Econ J. (1921) 31:124–6. doi: 10.2307/2223319

[27] Matsumoto A Merlone U Szidarovszky F. Some notes on applying the Herfindahl-Hirschman index. Appl Econ Lett. (2012) 19:181–4. doi: 10.1080/13504851.2011.570705

[28] Milo R Shen-Orr S Itzkovitz S Kashtan N Chklovskii D Alon U. Network motifs: simple building blocks of complex networks. Science. (2002) 298:824–7. doi: 10.1126/science.298.5594.824, 12399590

[29] Biewen M. Bootstrap inference for inequality, mobility and poverty measurement. J Econ. (2002) 108:317–42. doi: 10.1016/s0304-4076(01)00138-5

[30] Kanbur R Zhang X. Which Regional Inequality? The Evolution of Rural–Urban and Inland–Coastal Inequality in China from 1983 to 1995. J Comp Econ. (1999) 27:686–701. doi: 10.1006/jcec.1999.1612

[31] Liu HM Jiang JY Yu LC Liu XP. The impact of hospital competition on healthcare quality: evidence from China's healthcare reform. Front Public Health. (2025) 13:14. doi: 10.3389/fpubh.2025.1543884, 40520308 PMC12162968

[32] Wang CL Li C Zhang AN Gu GX Yang SG. Healthcare inequities in Chinese megacities: the older adult population's accessibility to public hospitals in suburban Shanghai. Front Public Health. (2025) 13:18. doi: 10.3389/fpubh.2025.1700098, 41487631 PMC12756823

[33] Dong EH Xu J Sun XT Xu T Zhang LF Wang T. Differences in regional distribution and inequality in health-resource allocation on institutions, beds, and workforce: a longitudinal study in China. Arch Public Health. (2021) 79:78. doi: 10.1186/s13690-021-00597-1, 34001268 PMC8130126

[34] Tao ZL Cheng Y Zheng QJ Li GC. Measuring spatial accessibility to healthcare services with constraint of administrative boundary: a case study of Yanqing District, Beijing, China. nt J Equity Health. (2018) 17:12. doi: 10.1186/s12939-018-0720-5, 29334979 PMC5769485

[35] Crook H Horta M Michelson KA Graves JA. Performance of health care service area definitions for capturing variation in inpatient care and social determinants of health. Health Serv Res. (2024) 59:e14312. doi: 10.1111/1475-6773.14312, 38698467 PMC11249811

[36] Balia S Brau R Moro D. Choice of hospital and long-distances: evidence from Italy. Reg Sci Urban Econ. (2020) 81:103502. doi: 10.1016/j.regsciurbeco.2019.103502

[37] Jing RZ Tang J Song YP Ni CX. Enhancing health system efficiency in China: considering the interaction between use of primary care and the demand for secondary and tertiary care. Health Policy Plan. (2025) 40:876–88. doi: 10.1093/heapol/czaf047, 40799119 PMC12448839

[38] Wu BL Wu WB Wang X Huang WZ Luo ZN Li JF. Evaluating and enhancing the service capacity of secondary public hospitals in urban China: a multi-method empirical analysis based on Guangzhou (2019-2023). Front Health Serv. (2025) 5:13. doi: 10.3389/frhs.2025.1621018, 40575546 PMC12198218

